# Mass Production of *Lemna minor* and Its Amino Acid and Fatty Acid Profiles

**DOI:** 10.3389/fchem.2018.00479

**Published:** 2018-10-15

**Authors:** Rina Chakrabarti, William D. Clark, Jai Gopal Sharma, Ravi Kumar Goswami, Avanish Kumar Shrivastav, Douglas R. Tocher

**Affiliations:** ^1^Aqua Research Lab, Department of Zoology, University of Delhi, New Delhi, India; ^2^Institute of Aquaculture, Faculty of Natural Sciences, University of Stirling, Stirling, Scotland; ^3^Department of Biotechnology, Delhi Technological University, New Delhi, India

**Keywords:** *Lemna minor*, organic manure, proximate composition, amino acids, fatty acids

## Abstract

The surface floating duckweed *Lemna minor* (Lemnaceae) is a potential ingredient to replace the application of fish-meal in the aqua-feed. The culture technique of the duckweed was standardized in outdoor tanks and then applied in the pond. Three consecutive experiments were conducted in tanks (1.2 × 0.35 × 0.3 m). In experiment 1, four different manures were used. In manure 1 (organic manure, OM) and manure 3 (2x OM), cattle manure, poultry droppings, and mustard oil cake (1:1:1) were used; in manure 2 (inorganic fertilizer, IF), urea, potash, triple superphosphate were used; manure 4 (2x OM+IF) was a combination of manure 2 and manure 3. In experiment 2, manure 1 (OM) and manure 2 (IF) were used, and manure 3 (OM+IF) was a combination of both manures. In experiment 3, OM and IF were selected. In pond (20 × 10 × 0.5 m), OM was applied. Fresh duckweed was seeded after 5 days of manure application. In experiments 1 and 3, total production was significantly (*P* < 0.05) higher in OM compared to other treatments. In experiment 2, there was no significant (*P* > 0.05) difference in production between OM and IF. In pond, relative growth rate (RGR) of duckweed ranged from 0.422 to 0.073 g/g/day and total production was 702.5 Kg/ha/month (dry weight). Protein, lipid, and ash contents were higher in duckweed cultured in OM compared to IF. The duckweed was a rich source of essential (39.20%), non-essential (53.64%), and non-proteinogenic (7.13%) amino acids. Among essential amino acids, leucine, isoleucine, and valine constituted 48.67%. Glutamic acid was 25.87% of total non-essential amino acids. Citrulline, hydroxiproline, taurine, etc. were found in the duckweed. The fatty acid composition was dominated by PUFA, 60–63% of total fatty acids, largely α-linolenic acid (LNA, 18:3n-3) at around 41 to 47% and linoleic acid (LA, 18:2n-6) at 17–18%. The nutritional value of duckweeds and their production potential in the pond conditions were evaluated. Duckweed biomass may thus be used to replace commercial fish-meal that is currently used in aquaculture.

## Introduction

The surface floating macrophyte duckweed *Lemna* is the largest genus of the family Lemnaceae. They are abundant in the tropical and subtropical countries; growing profusely in still, nutrient-rich small ponds, ditches, and swamps or in slowly moving water bodies. The entire plant body consists of metabolically active non-structural tissue (Wolverton and McDonald, 1980) and the low fiber content of the plant has a beneficial impact on digestibility when used in animal feed. Duckweed grows on water with relatively high levels of N, P, and K and concentrates the minerals and synthesizes protein. The reported presence of various essential (arginine, histidine, isoleucine, leucine, lysine, methionine, phenylalanine, threonine, valine, tyrosine) and non-essential amino acids (FAO, 2001), poly-unsaturated fatty acids (Yan et al., 2013), β-carotene, and xanthophylls has made *Lemna* spp. a potential feed source for livestock (Skillicorn et al., 1993; Leng et al., 1995). In Taiwan, duckweeds are used as food for pig and poultry (FAO, 2001). In fish feed, *Lemna* spp. are usually used in fresh state. In recent years, there is a growing interest in this free floating macrophyte in the aqua-feed industry (for production of pelleted diets) to replace the protein-rich and costly fish-meal. Chakrabarti (2017) reported the production potential of duckweeds in freshwater bodies. Duckweeds are also used for treatment of waste water (Culley and Epps, 1973; Sutton and Ornes, 1975, 1977) and production of bio-fuel (Jarvis et al., 1988; Zhao et al., 2012, 2014, 2015a,b).

Protein plays a significant role in fish nutrition. Fish-meal is one of the commonly used protein-rich ingredients in the aqua-feed industry. Non-availability of quality fish meal and competition for the same resources with the terrestrial live-stock industry has made aquaculturists search for economically viable alternative protein sources. The alternative protein source (to fish meal) should be available in the required amount, cost-effective and preferably non-conventional to avoid competition with other uses and industries. The amino acid profile of the ingredient should meet the nutritional requirement of the cultivable species and prepared feed should be palatable and digestible to the fish. Digestibility test of duckweeds in carps and tilapia showed promising results (Hassan and Chakrabarti, 2009). Sharma et al. (2016) reported that the protein content of *Lemna minor* was 39.75 ± 0.47% and that digestibility of this plant protein for rohu *Labeo rohita* and common carp *Cyprinus carpio* was high as determined by an *in vitro* digestibility study.

The application of *Lemna* spp. as potential aqua-feed ingredients requires continuous production. Sustainable production of this plant requires an understanding of its nutritional and environmental requirements. The nutritional value of a plant also depends on the culture medium. The growth rate of duckweed clones in different natural (Rejmankova, 1975) and laboratory (Landolt, 1957) conditions varied. Many studies showed the production of *Lemna* spp. in domestic waste water (Zirschky and Reed, 1988), septage-fed ponds (Edwards et al., 1990, 1992), and effluent water (Vroon and Weller, 1995). The production of *Lemna* spp. in clean water with a known manuring schedule is required for commercial aqua-feed production. Few studies have been conducted to find the best balance of nutrients that may provide maximum growth of duckweed (FAO, 1989), especially for *Lemna* spp. The requirement to fertilize duckweeds depends on the source of the water. Rainwater collected in ponds may need a balanced NPK application. In Bangladesh, inorganic fertilizers (IFs, urea, triple superphosphate, and potash) were used for the production of duckweeds (DWRP, 1998). Hassan and Chakrabarti (2009) suggested a wide range of organic waste materials *viz*. animal manure, kitchen wastes, wastes from a wide range of food processing plants, biogas effluents, etc. for the production of duckweeds. A periodic supply of nutrients helped to avoid nutrient deficiency in the culture systems (Sutton and Ornes, 1975; Said et al., 1979). A direct relationship was found between the crude protein content of duckweed and the nitrogen content of the culture system. Although many species survive at extreme temperature, warm and sunny conditions are preferable for faster growth of the plants (Skillicorn et al., 1993). The distribution of various members of duckweed has been influenced by the microclimatic factors such as light intensity, salinity, and regional temperature (Landolt, 1986). The growth of duckweed is largely a function of environmental temperature and light, nutrient status of the culture medium and the degree of crowding of the plants (Hassan and Chakrabarti, 2009).

The present investigation aimed to develop a suitable culture technique for the production of *L. minor* in a sustainable manner. In our earlier study, it was found that the application of organic manures (OM) viz. cattle manure, poultry wastes, and mustard oil-cake was very effective in the mass production of live food organisms (Srivastava et al., 2006). Application of these manures helped in the large scale production of zooplankton in the outdoor facility (Chakrabarti and Sharma, 2008). These manures are easily available. Therefore, in the present study, these OMs were selected along with the other IFs to evaluate their effect on the production of duckweed. The culture technique was first standardized in a small outdoor facility under controlled conditions using various organic and IFs. The best method was then adopted in pond conditions to evaluate the large scale production potential of the macrophyte. The nutritional value of the produced plant was determined to evaluate its suitability as a potential feed ingredient for the aqua-feed industry.

## Results

### Culture of *L. minor* in outdoor tanks

#### Water quality

**Figure 1:**
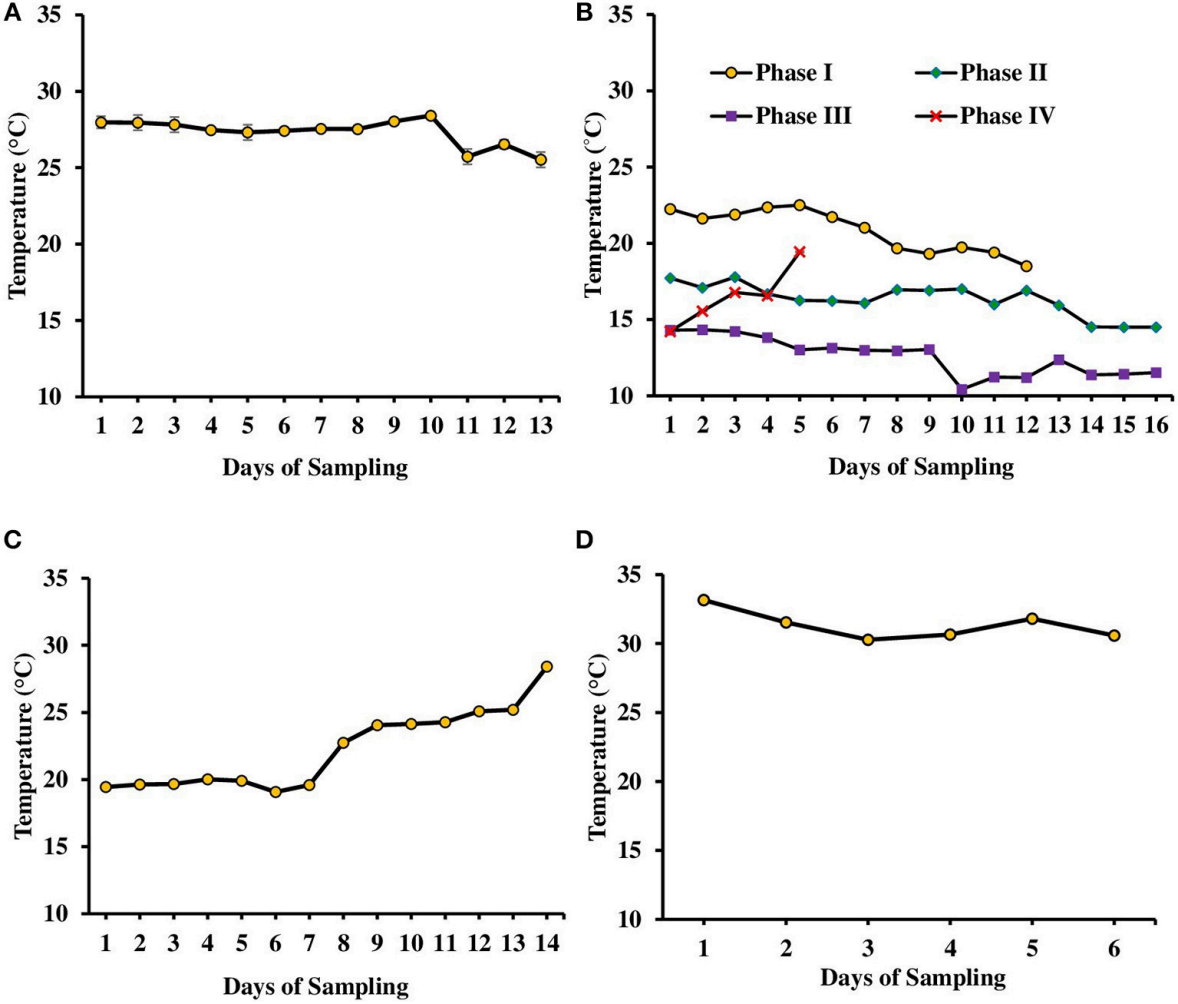
Variation in water temperature found in tanks (experiments 1–3) and ponds during various days of culture of *L. minor*. **(A)** Experiment 1. **(B)** Experiment 2 has been divided in four phases depending on temperature—Phase I (October–November 2016), Phase II (November–December 2016), Phase III (December 2016–January 2017), and Phase IV (January–February 2017). The water temperature ranged from 22.5 to 18.5, 17.7 to 14.5, 14.3 to 11.2, and 14.2 to 19.4°C in first, second, third, and fourth phases, respectively. **(C)** Experiment 3 and **(D)** in ponds. There was no significant difference in temperature among various treatments of each experiment. Therefore, data for individual day of all treatments are presented as Mean ± SE. Data were collected at 9.00 a.m.

**Figure 2:**
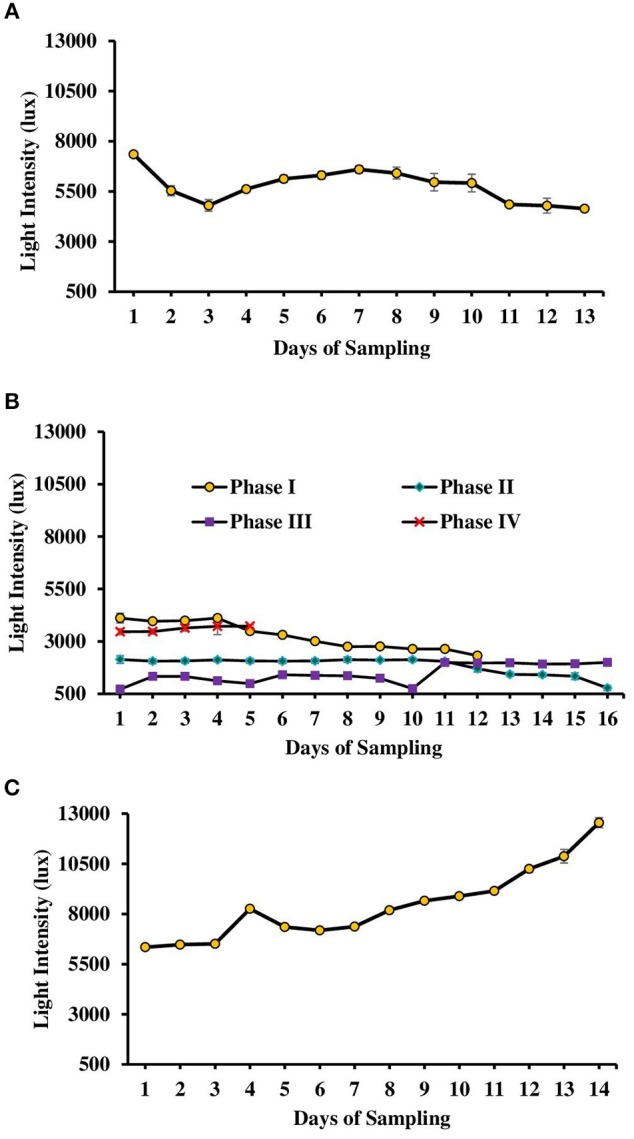
Light intensity monitored during various days of culture of *L. minor* in tanks. **(A)** Experiment 1. **(B)** Experiment 2 has been divided in four phases depending on temperature—Phase I (October–November 2016), Phase II (November–December 2016), Phase III (December 2016–January 2017), and Phase IV (January–February 2017). **(C)** Experiment 3. There was no significant difference in light intensity among various treatments of each experiment. Therefore, data for individual day of all treatments were presented as Mean ± SE. Data were collected at 9.00 a.m. (Data for experiment 4 has not given.).

**Figure 3:**
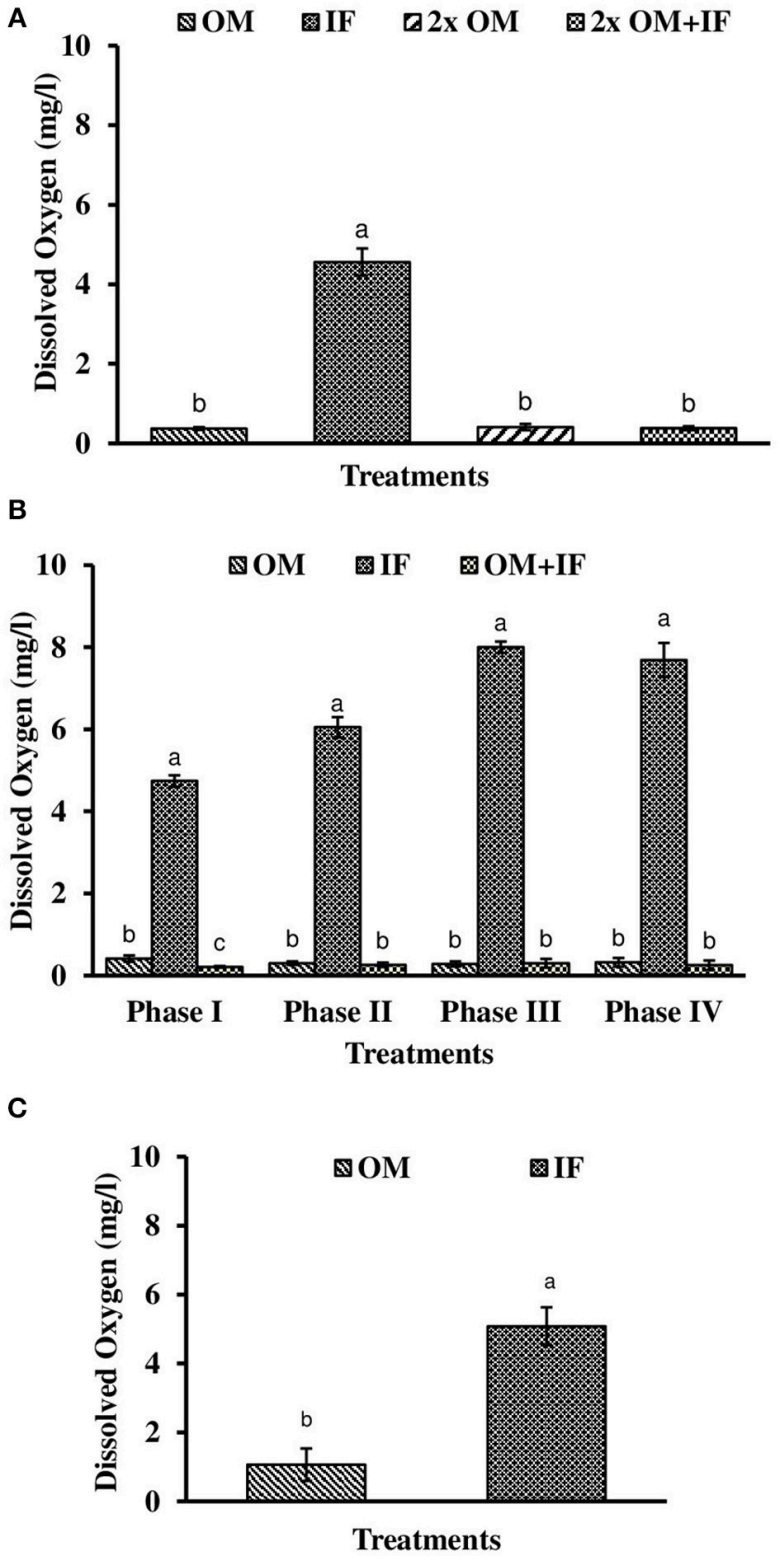
Dissolved oxygen levels (in parenthesis) of water found in various treatments of tank experiments of *L. minor*. **(A)** Experiment 1, **(B)** experiment 2 (Phase I, Phase II, Phase III, and Phase IV), and **(C)** experiment 3. In each treatment (three replicates), average values of 13, 49 (Phase I−12, Phase II and III−16 each, and Phase IV−5 days), and 14 days of sampling for experiments 1, 2, and 3, respectively, were calculated. Data were presented as Mean ± SE. Bars with different superscripts were significantly (*P* < 0.05) different. Data were collected at 9.00 a.m. OM, organic manures; IF, inorganic fertilizers; 2x OM, double dose of organic manures.

**Figure 4:**
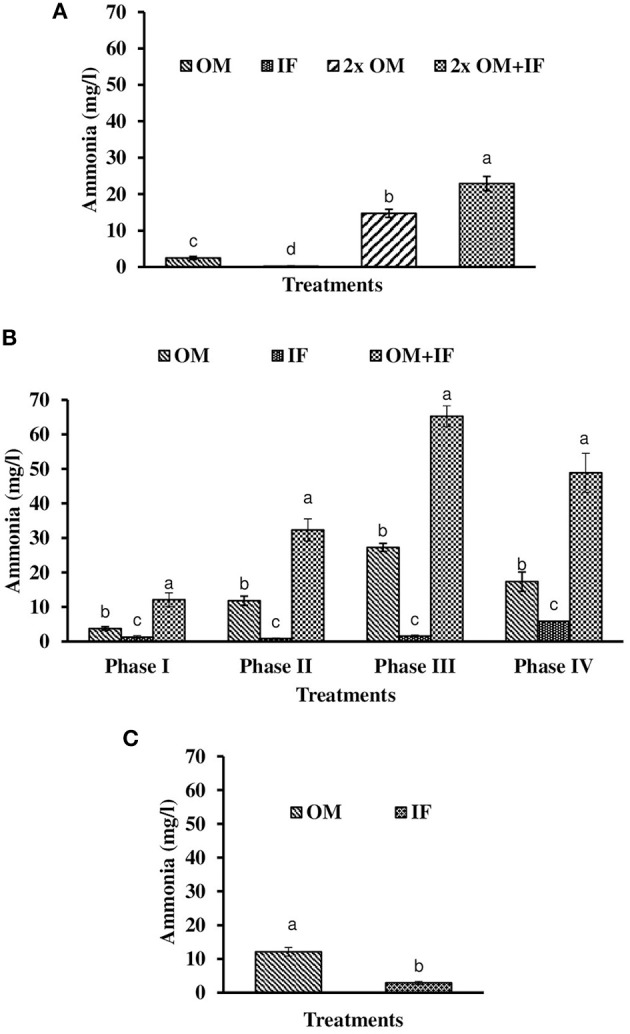
Ammonia levels (in parenthesis) of water found in various treatments of tank experiments of *L. minor*. **(A)** Experiment 1, **(B)** experiment 2 (Phase I, Phase II, Phase III, and Phase IV), and **(C)** experiment 3. In each treatment (three replicates), average values of 13, 49 (Phase I−12, Phase II and III−16 each, and Phase IV−5 days), and 14 days of sampling for experiments 1, 2, and 3, respectively, were calculated. Data were presented as Mean ± SE. Bars with different superscripts were significantly (*P* < 0.05) different. Data were collected at 9.00 a.m. OM, organic manures; IF, inorganic fertilizers; 2x OM, double dose of organic manures.

**Figure 5:**
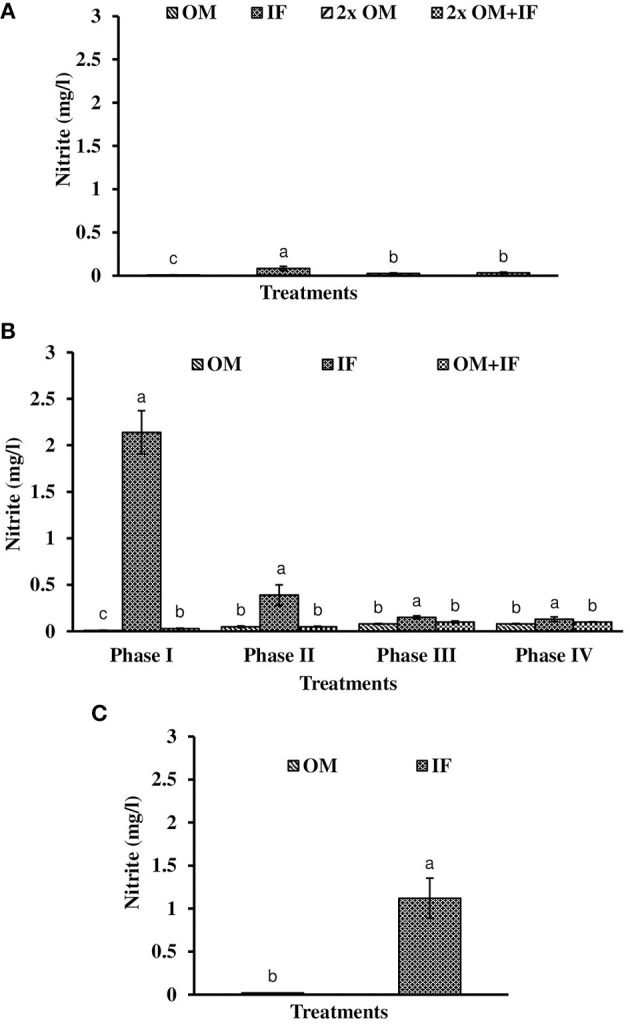
Nitrite levels (in parenthesis) of water found in various treatments of tank experiments of *L. minor*. **(A)** Experiment 1, **(B)** experiment 2 (Phase I, Phase II, Phase III, and Phase IV), and **(C)** experiment 3. In each treatment (three replicates), average values of 13, 49 (Phase I−12, Phase II and III−16 each, and Phase IV−5 days), and 14 days of sampling for experiments 1, 2, and 3, respectively, were calculated. Data were presented as Mean ± SE. Bars with different superscripts were significantly (*P* < 0.05) different. Data were collected at 9.00 a.m. OM, organic manures; IF, inorganic fertilizers; 2x OM, double dose of organic manures.

**Figure 6:**
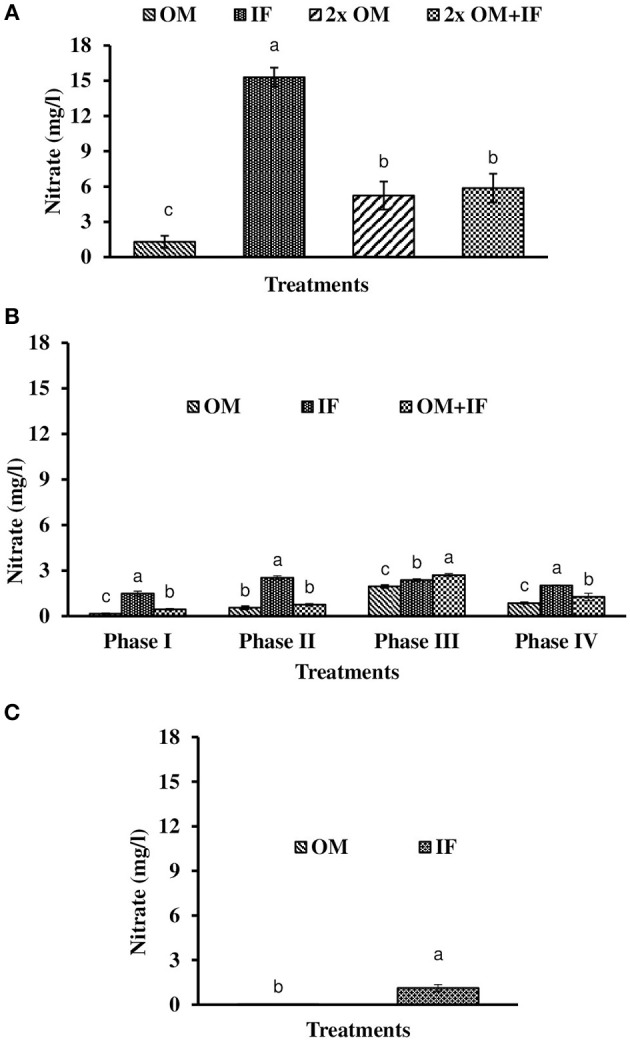
Nitrate levels (in parenthesis) of water found in various treatments of tank experiments of *L. minor*. **(A)** Experiment 1, **(B)** experiment 2 (Phase I, Phase II, Phase III, and Phase IV), and **(C)** experiment 3. In each treatment (three replicates), average values of 13, 49 (Phase I−12, Phase II and III−16 each, and Phase IV−5 days), and 14 days of sampling for experiments 1, 2, and 3, respectively, were calculated. Data were presented as Mean ± SE. Bars with different superscripts were significantly (*P* < 0.05) different. Data were collected at 9.00 a.m. OM, organic manures; IF, inorganic fertilizers; 2x OM, double dose of organic manures.

**Figure 7:**
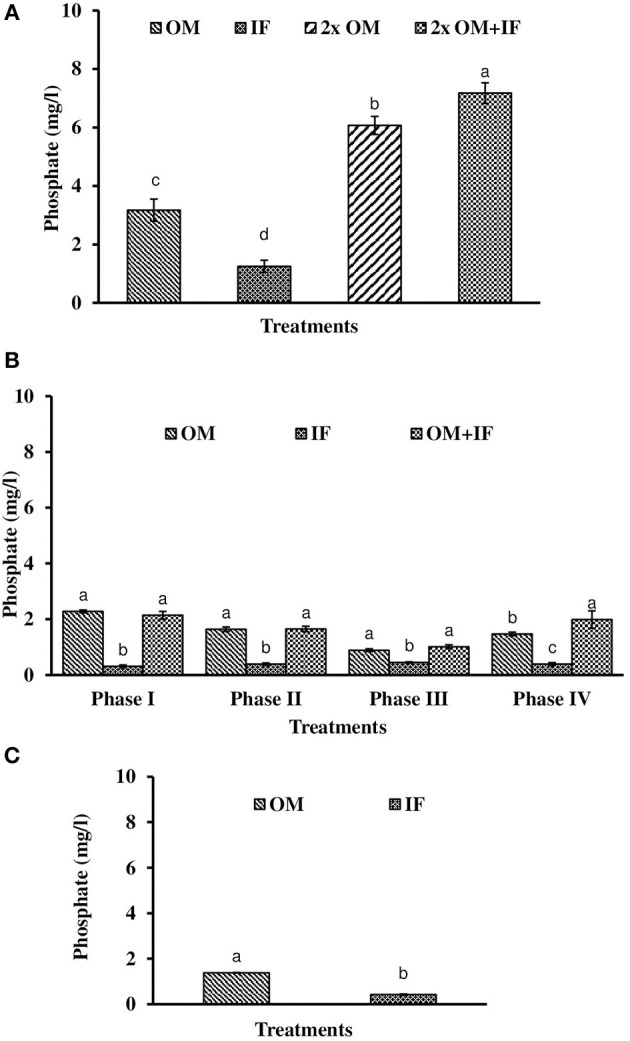
Phosphate levels (in parenthesis) of water found in various treatments of tank experiments of *L. minor*. **(A)** Experiment 1, **(B)** experiment 2 (Phase I, Phase II, Phase III, and Phase IV), and **(C)** experiment 3. In each treatment (three replicates), average values of 13, 49 (Phase I−12, Phase II and III−16 each, and Phase IV−5 days), and 14 days of sampling for experiments 1, 2, and 3, respectively, were calculated. Data were presented as Mean ± SE. Bars with different superscripts were significantly (*P* < 0.05) different. Data were collected at 9.00 a.m. OM, organic manures; IF, inorganic fertilizers; 2x OM, double dose of organic manures.

**Figure 8:**
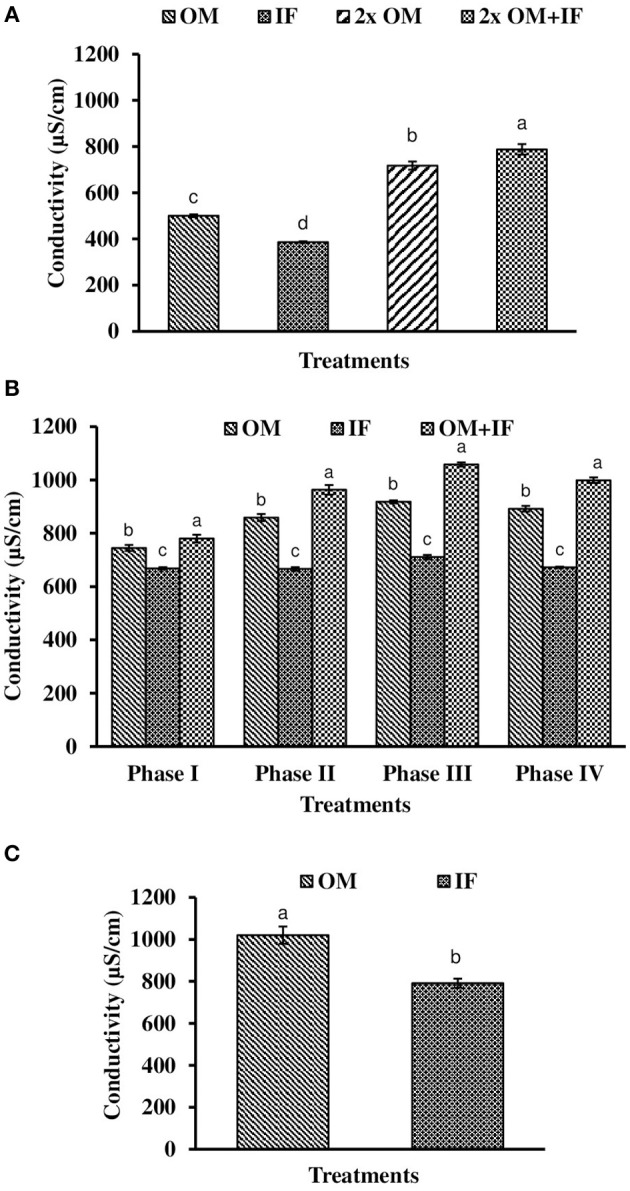
Conductivity of water (in parenthesis) found in various treatments of tank experiments of *L. minor*. **(A)** Experiment 1, **(B)** experiment 2 (Phase I, Phase II, Phase III, and Phase IV), and **(C)** experiment 3. In each treatment (three replicates), average values of 13, 49 (Phase I−12, Phase II and III−16 each, and Phase IV−5 days), and 14 days of sampling for experiments 1, 2, and 3, respectively, were calculated. Data were presented as Mean ± SE. Bars with different superscripts are significantly (*P* < 0.05) different. Data were collected at 9.00 a.m. OM, organic manures; IF, inorganic fertilizers; 2x OM, double dose of organic manures.

Three consecutive experiments were conducted in outdoor tanks to generate the baseline data for the production of duckweed *L. minor* in the pond conditions. Four, three, and two different manures were used in experiments 1, 2, and 3, respectively. In the pond experiment, only OM were applied for the production of *L. minor*. In experiment 1, water temperature ranged from 28.0 ± 0.4 to 25.5 ± 0.3°C in various treatments during September–October 2016. The intensity of light was recorded as 7,353 ± 138 to 4,642 ± 114 lux during this period in different culture tanks. There was no significant (*P* > 0.05) difference in water temperature (Figure 1A) and light intensity (Figure 2A) among various culture tanks. Water temperature and light intensity were higher at the beginning of the study and gradually reduced. The pH of water ranged from 7.10 to 7.41, 7.30 to 7.80, 6.96 to 7.35, and 6.99 to 7.52 in manures 1, 2, 3, and 4, respectively, throughout the study period. Dissolved oxygen level was significantly (*P* < 0.05) higher in the culture system fertilized with IFs compared to the other treatments throughout the study period. Dissolved oxygen levels in other treatments were always less than one (Figure 3A). Ammonia (NH_3_) level was significantly (*P* < 0.05) higher in 2x OM+IF treatment compared to the others throughout the study period (Figure 4A). Among these four treatments, lowest ammonia level was found in IF. Ammonia level ranged from 0.585 to 4.65, 0.03 to 0.51, 8.4 to 22.6, and 15.65 to 42.97 mg/l in OM, IF, 2x OM, and 2x OM+IF, respectively, throughout the study period. Nitrite level was significantly (*P* < 0.05) higher in IF compared to the other treatments (Figure 5A). Highest level was recorded on day-1 of study in this treatment. Nitrite levels were 0.008 ± 0.002, 0.084 ± 0.024, 0.025 ± 0.008, and 0.033 ± 0.009 mg/l in OM, IF, 2x OM, and 2x OM+IF, respectively. Nitrate level was significantly (*P* < 0.05) higher in IF compared to the other treatments (Figure 6A). Nitrate levels were 1.30 ± 0.52, 15.30 ± 0.80, 5.23 ± 1.20, and 5.87 ± 1.22 mg/l in OM, IF, 2x OM, and 2x OM+IF, respectively. This showed the rate of nitrification among various treatments. Phosphate level was significantly (*P* < 0.05) higher in 2x OM+IF compared to the other treatments. This group was followed by 2x OM, OM, and minimum level was found in IF (Figure 7A). Highest level of phosphate was recorded on day-1 of study compared to the other days regardless of treatments. Conductivity was significantly (*P* < 0.05) higher in 2x OM+IF compared to the other treatments (Figure 8A). This group was followed by 2x OM, OM, and minimum level was found in IF.

The second experiment was conducted during October 2016–February 2017. In this experiment, seasonal effect on water quality and *L. minor* production was recorded (Figure 1B). Depending on the range of water temperature and light intensity the whole study period was divided into four phases. In the first phase (October–November) of the study, water temperature ranged from 22.5 to 18.5°C and then it reduced. Water temperature ranged from 17.7 to 14.5, 14.3 to 11.2, and 14.2 to 19.4°C in second (November–December), third (December–January), and fourth (January–February) phases, respectively. Light intensity also varied significantly in four different phases (Figure 2B). Light intensity ranged from 4,111 ± 232 to 2,322 ± 130, 2,138 ± 178 to 781 ± 122, 718 ± 37 to 1,999 ± 34, and 3,463 ± 114 to 3,728 ± 57 lux in phase I, phase II, phase III, and phase IV, respectively. There was no significant (*P* > 0.05) difference in temperature and light intensity in various treatments throughout the study period. In OM, pH ranged from 7.19 to 7.60, 7.23 to 7.88, 7.52 to 7.91, and 7.66 to 8.13 in the first, second, third, and fourth phases, respectively. In IF, pH ranged from 7.50 to 7.86, 7.63 to 8.07, 7.68 to 8.05, and 7.60 to 8.09 in the first, second, third, and fourth phases, respectively. In OM+IF, pH ranged from 7.30 to 7.67, 7.32 to 7.86, 7.47 to 7.94, and 7.80 to 7.90 in the first, second, third, and fourth phases, respectively.

Dissolved oxygen level was significantly (*P* < 0.05) higher in IF compared to the other two treatments throughout the study period (Figure 3B). Dissolved oxygen level ranged from 4.7 to 8.7 mg/l in various days of study in IF. Dissolved oxygen level was < 1 mg/l in most of the days of study in OM and OM+IF. Ammonia (NH_3_) level was significantly (*P* < 0.05) higher in OM+IF compared to the other two treatments in all four phases of the study (Figure 4B). In OM and OM+IF, highest ammonia level was found at phase III. In OM, ammonia level ranged 1.74 to 5.86, 4.61 to 21.06, 16.36 to 31.56, and 14.23 to 22.93 mg/l in the first, second, third, and fourth phases, respectively. In IF, ammonia level ranged from 0.20 to 2.88, 0.11 to 2.18, 0.57 to 5.37, and 5.80 to 5.92 mg/l in the first, second, third, and fourth phases, respectively. In OM+IF, ammonia level ranged from 5.42 to 19.96, 9.86 to 51.6, 47.70 to 88.5, and 42.96 to 60.16 mg/l in the first, second, third, and fourth phases, respectively. Nitrite level was significantly (*P* < 0.05) higher in IF compared to the other two treatments throughout the study period (Figure 5B). Nitrite levels were 0.01 to 0.08, 2.14 to 0.1, and 0.03 to 0.13 mg/l in OM, IF, and OM+IF, respectively, in all four phases. Nitrate level was significantly (*P* < 0.05) higher in IF compared to the other two treatments in first and second phases (Figure 6B). In OM+IF, significantly (*P* < 0.05) higher nitrate level was found in the third phase of the study. Nitrate levels were 0.16 to 1.95, 1.48 to 2.53, and 0.44 to 2.70 mg/l in OM, IF, and OM+IF, respectively, throughout the study period. Phosphate level was significantly (*P* < 0.05) lower in IF compared to the other two treatments throughout the study period (Figure 7B). In OM, phosphate level ranged 2.03 to 2.54, 1.22 to 2.22, 0.45 to 1.21, and 1.35 to 1.60 mg/l in the first, second, third, and fourth phases, respectively. In IF, phosphate level ranged from 0.15 to 0.42, 0.22 to 0.65, 0.30 to 0.55, and 0.28 to 0.45 mg/l in the first, second, third, and fourth phases, respectively. Conductivity was significantly (*P* < 0.05) higher in OM+IF compared to the other two treatments throughout the study period (Figure 8B). This group was followed by OM and IF.

The third experiment was conducted during February–April 2017. In this experiment, water temperature was minimum at the beginning and gradually increased ranging from 19.43 to 28.42°C. Light intensity also showed an increasing trend ranging from 6,341 ± 10 to 12,550 ± 283 lux throughout the study period. There was no significant (*P* > 0.05) difference in temperature (Figure 1C) and light intensity (Figure 2C) between the two treatments throughout the study period. The pH of the water ranged from 7.41 to 7.83 and 7.60 to 9.14 in OM and IF, respectively, during the study period. Dissolved oxygen level was significantly (*P* < 0.05) higher in IF compared to the OM throughout the study period. Dissolved oxygen level ranged from 1.27 to 0.12 mg/l in various days of study in OM. Dissolved oxygen level was always < 1 mg/l in OM, except on the first day after manure application (Figure 3C).

Ammonia (NH_3_) level was significantly (*P* < 0.05) higher in OM compared to IF throughout the study period (Figure 4C). Ammonia level ranged from 7.09 to 17.4 and 0.27 to 5.78 mg/l in OM and IF, respectively. Nitrite (Figure 5C) and nitrate (Figure 5C) levels were significantly (*P* < 0.05) higher in IF compared to the OM throughout the study period. Nitrite level ranged from 0.008 to 0.04 and 0.11 to 2.66 mg/l in OM and IF, respectively, during the study period. Nitrate level ranged from 0.05 to 1.61 and 1.13 to 4.32 mg/l in OM and IF (Figure 6C), respectively. Phosphate level was significantly (*P* < 0.05) higher in OM compared to IF throughout the culture period. Phosphate level ranged 1.29 to 1.65 and 0.24 to 0.50 mg/l in OM and IF, respectively, throughout the study period (Figure 7C). Conductivity was significantly (*P* < 0.05) higher in OM compared to IF (Figure 8C).

#### Relative growth rate (RGR) and production

**Figure 9:**
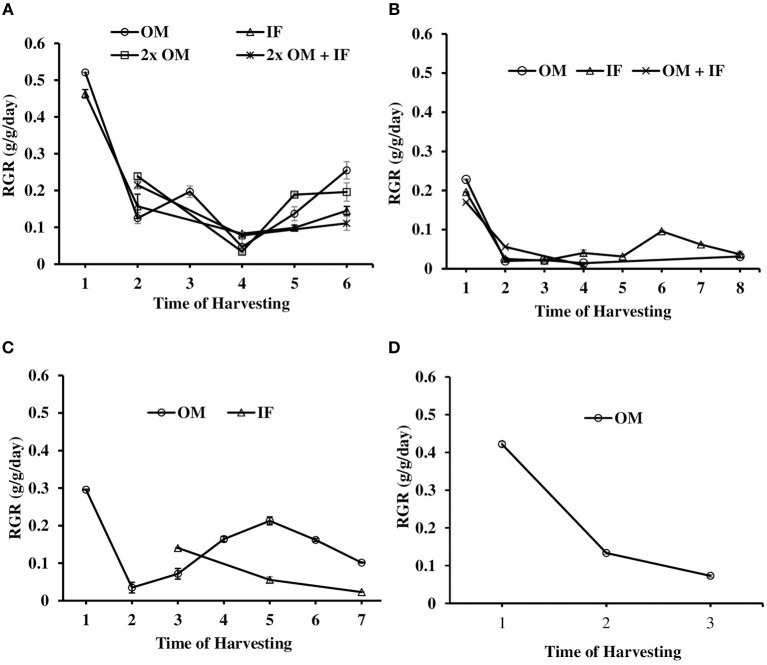
Relative growth rate (RGR) of *L. minor* found in **(A)** experiment 1, **(B)** experiment 2, **(C)** experiment 3, and **(D)** ponds during various days of culture. In experiment 2, harvesting pattern was as follows: Phase I—first harvest, Phase II—second, third, and fourth harvests, Phase III—fifth, sixth, and seventh harvests, Phase IV—eighth harvest. In experiment 3, there was no harvesting in IF on 1, 2, 4, and 6 sampling. OM, organic manures; IF, inorganic fertilizers; 2x OM, double dose of organic manures.

The relative growth rate (RGR) of *L. minor* varied among different treatments in all three experiments. In experiment 1, *L. minor* was harvested 6, 5, 4, and 3 times in OM, IF, 2x OM, and 2x OM+IF, respectively, during 30 days of culture period. The RGR was always highest at first harvest regardless of treatments (Figures 9A–C). In experiment 1, RGR ranged from 0.521 to 0.047, 0.463 to 0.083, 0.239 to 0.034, and 0.215 to 0.078 g/g/day in OM, IF, 2x OM and 2x OM+IF, respectively, in various days of sampling. In experiment 2, plants were harvested 5, 8, and 3 times from OM, IF, and OM+IF, respectively. The RGR ranged from 0.23 to 0.014, 0.196 to 0.021, and 0.169 to 0.056 g/g/day in OM, IF, and OM+IF, respectively, in various days of sampling. In experiment 3, plants were harvested 7 and 3 times from OM and IF, respectively. The RGR ranged from 0.30 to 0.035 and 0.140 to 0.023 g/g/day in OM and IF in various days of sampling.

#### Production of *L. minor* in outdoor tanks

**Figure 10:**
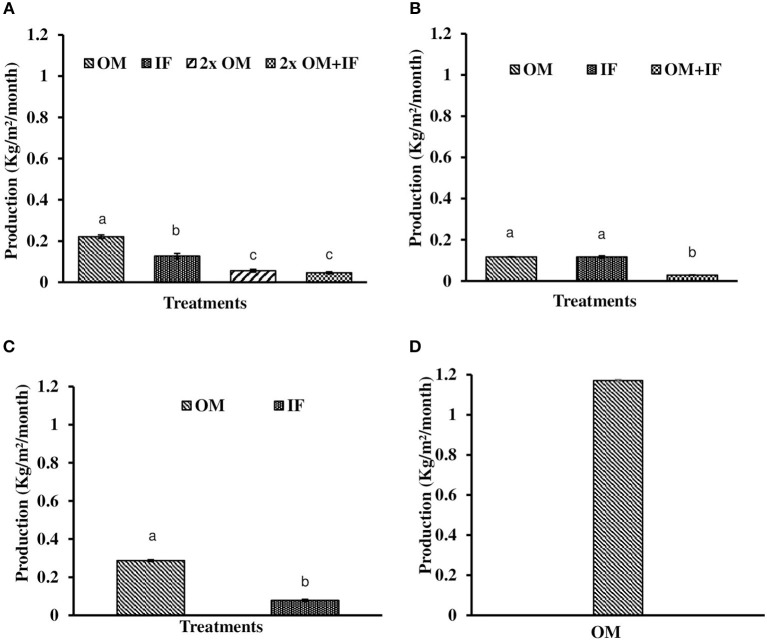
Total production of *L. minor* found in **(A)** experiment 1, **(B)** experiment 2, **(C)** experiment 3, and **(D)** ponds. Bars with different superscripts are significantly (*P* < 0.05) different (*n* = 3). OM, organic manures; IF, inorganic fertilizers; 2x OM, double dose of organic manures.

In all these study, macrophytes were harvested when the surface area of the tanks were filled with macrophytes. There was difference in harvesting time in various treatments due to differences in the growth of plants. In all these treatments, 50% of the total biomass was harvested at each harvest, except the final one. In experiment 1, production of macrophytes was significantly (*P* < 0.05) higher in OM compared to the other treatments (Figure 10A). Lowest production was recorded in 2x OM+IF throughout the study period.

In experiment 2, *L. minor* production was significantly (*P* < 0.05) higher in OM compared to the other two treatments in first and second phases. In third phase, macrophyte was harvested thrice from IF. There was no harvesting from OM as the growth of duckweed was very less and it was not covering the whole surface area of the tank. Plants were harvested only when they covered the whole water body. The growth of macrophytes was very poor in OM+IF in the third phase. There was no survival of plants in this treatment. In fourth phase, *L. minor* production was significantly (*P* < 0.05) higher in OM compared to IF. Total production was significantly (*P* < 0.05) higher in OM and IF compared to OM+IF treatment. There was no significant (*P* > 0.05) difference in total production between OM and IF (Figure 10B).

In experiment 3, *L. minor* production was significantly (*P* < 0.05) higher in OM compared to the IF (Figure 10C). In OM, macrophyte was first harvested after 14 days of inoculation of plants when the tank was totally covered with macrophytes. In IF, tanks were filled with macrophytes after 28 days of inoculation and then plants were harvested. The type of manures influenced the growth of macrophytes.

### Culture of *L. minor* in ponds

#### Water quality

This experiment was conducted in cemented ponds during July–August 2017. The variations in water quality parameters reflected the seasonal variations as well as effect of manure application. Water temperature and pH ranged from 33.15 to 30.27°C and 7.32 to 8.04, respectively, throughout the study period. Water temperature was higher in July and gradually it decreased (Figure 1D). Dissolved oxygen level ranged from 1.04 to 3.57 mg/l on various days of study. Dissolved oxygen level was higher at the beginning of the experiment; the level decreased after the application of manures and with the growth of macrophytes as it covered the surface area of the ponds. Ammonia, nitrite and nitrate levels ranged from 5.02 to 10.57, 0.003 to 0.12, and 0.23 to 2.44 mg/l, respectively. Phosphate level ranged 1.15 to 1.70 mg/l during the study period (Table 1). Conductivity ranged from 1,022 to 1,351 μS/cm throughout the culture period of duckweed. Ammonia, nitrite, nitrate, and phosphate levels varied with the days of manure application.

**Table 1:** Culture conditions of *L. minor* in ponds.

**Parameter**	**Range**	**Mean ±SE**
Temperature (°C)	30.27–33.15	31.32 ± 1.0
pH	7.32–8.04	–
Dissolved oxygen (mg/l)	1.04–3.57	2.25 ± 0.52
Ammonia (mg/l)	5.02–10.57	3.25 ± 0.7
Nitrite (mg/l)	0.003–0.12	0.045 ± 0.01
Nitrate (mg/l)	0.23–2.44	1.13 ± 0.01
Phosphate (mg/l)	1.15–1.70	1.52 ± 0.07
Conductivity (μS/cm)	1,022–1,351	*1, 161*±1.7

*The average values (three replicates) of 6 days of sampling were presented as Mean ± SE*.

#### Relative growth rate (RGR) and production

The RGRs of *L. minor* were 0.422, 0.133, and 0.073 g/g/day in first, second, and third harvest times, respectively (Figure 9D). The duckweed was first harvested after 10 days of introduction. Macrophyte was harvested thrice during 1 month culture period. Fifty percent macrophyte was harvested at the time of first and second harvesting and all plants were collected at the time of third harvest. The production of *L. minor* was 1.17 ± 0.005 Kg/m^2^/month (Figure 10D). Total production of duckweed in the pond was 702.5 Kg/ha/month (dry weight).

#### Composition of *L. minor*

Proximate composition analysis of duckweed showed that there was difference between the macrophytes cultured with OM and IF. Protein, lipid, and ash contents were significantly (*P* < 0.05) higher in macrophytes cultured in OM compared to IF. Balancing these higher levels, carbohydrate content was lower in macrophytes cultured in OM compared to IF (Table 2).

**Table 2:** Proximate composition of *L. minor* (% of dry weight) grown in the tanks.

	**Organic manure (OM)**	**Inorganic fertilizer (IF)**
Protein	36.07 ± 0.18	27.12 ± 0.40^*^
Lipid	8.45 ± 0.61	7.15 ± 0.06
Ash	21.41 ± 0.20	19.42 ± 0.30^*^
Carbohydrate	34.07 ± 0.36	46.31 ± 0.74^*^

* *Denotes significant difference (P < 0.05)*.

The amino acid profile of duckweed cultured in OM showed some interesting results (Table 3). The essential (39.20%), non-essential (53.64%), and non-proteinogenic (7.13%) amino acids were present in duckweed. All essential amino acids viz. histidine, isoleucine, leucine, lysine, methionine, phenylalanine, threonine, tryptophan, and valine were found in adequate quantity. Leucine, isoleucine, and valine consisted 48.67% of the essential amino acids. Among non-essential amino acids, glutamic acid was 25.87%. Several non-proteinogenic amino acids viz. citrulline, hydroxiproline, taurine etc. were also present in the duckweed. The amino acid profile of duckweed cultured in IF was not provided.

**Table 3:** Amino acid profile of *L. minor* cultured with organic manures (OM).

**Amino acids**	**Concentration (g/100 g)**
**ESSENTIAL**	
Histidine (His)	0.894 ± 0.011
Isoleucine (Ile)	2.043 ± 0.064
Leucine (Lue)	4.132 ± 0.046
Lysine (Lys)	2.683 ± 0.161
Methionine (Met)	0.859 ± 0.014
Phenylalanine (Phe)	2.571 ± 0.034
Threonine (Thr)	1.924 ± 0.138
Tryptophan (Trp)	0.365 ± 0.010
Valine (Val)	2.664 ± 0.096
**NON-ESSENTIAL**	
Alanine (Ala)	2.882 ± 0.041
Arginine (Arg)	3.060 ± 0.045
Asparatate (Asp)	3.714 ± 0.372
Cysteine (Cys)	0.381 ± 0.032
Glutamic Acid (Glu)	6.427 ± 0.102
Glycine (Gly)	2.861 ± 0.031
Proline (Pro)	1.248 ± 0.035
Serine (Ser)	2.348 ± 0.333
Tyrosine (Tyr)	1.905 ± 0.125
**NON-PROTEINOGENIC**	
Phosphoserine (p-Ser)	0.578 ± 0.000
Taurine (Tau)	0.041 ± 0.015
Phospho ethanol amine (PEA)	0.023 ± 0.006
Sarcosine (Sar)	0.097 ± 0.004
α Amino adipic acid (a-AAA)	0.045 ± 0.013
α Amino-n-butaric acid (a-ABA)	0.150 ± 0.012
Cystathionine (Cysthi)	0.093 ± 0.019
β-Alanine (b-Ala)	0.111 ± 0.020
β-Amino isobutyric acid (b-AiBA)	0.971 ± 0.271
γ-Amino-n-butyric acid (g-ABA)	0.405 ± 0.014
Ethanol amine (EOHNH_2_)	0.146 ± 0.004
Hydroxylysine (Hylys)	0.058 ± 0.007
Ornithine (Orn)	0.014 ± 0.001
1 Methylhistidine (1 Mehis)	0.087 ± 0.003
3 Methylhistidine (3 Mehis)	0.117 ± 0.004
Carnosine (Car)	0.106 ± 0.001
Hydroxy proline (Hypro)	0.133 ± 0.015
Citrulline (Cit)	0.126 ± 0.002

The fatty acid composition of *L. minor* was dominated by PUFA, which accounted for 60–63% of total fatty acids, largely α-linolenic acid (LNA, 18:3n-3) at around 41–47% and linoleic acid (LA, 18:2n-6) at 17–18%, followed by saturated fatty acids (~23–26%) and monoenes (11–12%) (Table 4). As with proximate composition, fatty acid profile was also influenced by manures, with *L. minor* grown in OM having significantly higher proportions of LA, LNA, saturated, and monounsaturated fatty acids, and total PUFA when compared to the inorganic counterpart (IF). Due to the higher lipid content of *L. minor* grown in OM, all fatty acids were found in higher absolute amounts (mg/100 g dry mass) in macrophytes grown in OMs. Irrespective of manure, the *L. minor* lipid profile contained no long-chain PUFA (LC-PUFA) such as docosahexaenoic acid (DHA, 22:6n-3), although there was a trace of eicosapentaenoic acid (EPA, 20:5n-3).

**Table 4:** Fatty acid composition of *L. minor* as percentage of total fatty acids (%) or as mg fatty acids per 100 g dry weight (absolute).

	**Organic manure (OM)**		**Inorganic fertilizer (IF)**	
	**Percentage**	**Absolute**	**Percentage**	**Absolute**
14:0	1.10 ± 0.02	36.7 ± 3.3	1.14 ± 0.07	26.6 ± 1.0
15:0	0.31 ± 0.03	10.1 ± 0.4	0.36 ± 0.04	8.4 ± 0.22^*^
16:0	19.14 ± 0.03	634.8 ± 43.0	22.21 ± 4.32	516.7 ± 51.4
17:0	20.47 ± 0.11	79.5 ± 0.1	22.32 ± 2.49	77.7 ± 2.5
18:0	1.08 ± 0.01	35.7 ± 2.9	1.47 ± 0.57	33.9 ± 10.1
24:0	1.10 ± 0.00	36.4 ± 2.6	1.22 ± 0.17	28.4 ± 1.2
Total saturated	22.72 ± 0.03	753.7 ± 51.3	26.40 ± 5.17	613.9 ± 61.9
16:1n-9	5.60 ± 0.07	185.7 ± 10.6	5.36 ± 0.42	126.2 ± 21.8
16:1n-7	2.32 ± 0.02	77.0 ± 5.9	2.23 ± 0.12	52.4 ± 7.7
18:1n-9	2.15 ± 0.19	71.6 ± 11.3	2.88 ± 1.22	66.3 ± 22.3
18:1n-7	1.46 ± 0.03	48.4 ± 4.3	1.46 ± 0.04	34.3 ± 2.4
Total monoenes	11.53 ± 0.17	382.6 ± 32.2	11.93 ± 0.73	279.2 ± 9.7^*^
18:2n-6	16.88 ± 0.04	560.1 ± 40.1	18.07 ± 1.71	422.2 ± 0.6^*^
Total n-6 PUFA	16.88 ± 0.04	560.1 ± 40.1	18.07 ± 1.71	422.2 ± 0.6^*^
18:3n-3	46.35 ± 0.07	*1, 537.5*±108.9	41.24 ± 7.16	976.2 ± 260.9
20:5n-3	0.14 ± 0.02	4.5 ± 0.3	0.14 ± 0.02	3.6 ± 0.5
Total n-3 PUFA	46.49 ± 0.13	*1, 542.0*±102.6	41.38 ± 7.36	979.8 ± 265.9
Total DMA	2.37 ± 0.04	78.6 ± 4.1	2.23 ± 0.24	52.6 ± 10.7
Total PUFA	63.38 ± 0.10	*2, 102.1*±142.6	59.45 ± 5.66	*1, 402.0*±266.5
Total fatty acids		*3, 317.0*±230.3		*2, 347.6*±225.0

*Results: mean ± SD. DMA, dimethyl acetals; PUFA, polyunsaturated fatty acids*.

* *Denotes significant difference between OM and IF (P < 0.05)*.

## Discussion

In the present study, suitable manures and their dose for the production of *L. minor* was studied first in outdoor-tanks and then the best condition was adopted in ponds. The results of three consecutive studies in outdoor systems showed that duckweed production was influenced by the quality of manures and doses, and environmental factors. Both organic and IFs were used separately and in combinations for the production of *L. minor*. In experiments 1 and 3, the average RGR of *L. minor* was higher in OM compared to IF. The average RGR-values of duckweeds in OM were same in experiment 1 in outdoor tanks and in the pond experiment (0.21 g/g/day). In experiment 1 and 3, total macrophytes production (Figures 6A–C) was significantly higher in OM compared to the other treatments; whereas in experiment 2, there was no significant difference in total production between OM and IF. The growth of duckweed was affected by low temperature in OM in the third phase (i.e., fifth, sixth, and seventh harvests) of experiment 2. Reduced growth of duckweed affected the total production. In contrast, in the culture system treated with IFs the growth was continued in the cold condition (Figure 5B). Production of macrophytes in 2x OM and 2x OM+IF of experiment 1, and OM+IF of experiment 2 were negligible. For this reason these manures were not adopted for the production of macrophytes and less discussed in the present study. Higher doses of manures resulted in significantly (*P* < 0.05) higher levels of ammonia that affected the growth and production of macrophytes in these treatments. RGR was always higher at first harvest regardless of treatments. In experiment 1, RGR in OM during second harvest was lower compared to 2x OM and 2x OM+IF as this was the first harvest for these two latter treatments. There was no production in all treatments, except OM at the time of third harvesting. Therefore, in experiment 1, the RGR was lower in OM during fourth harvest compared to the other treatments. Similarly, in experiment 3, the first harvest of *L. minor* from IF, was the third harvest for OM. The poor growth rate of duckweed resulted in slow production and delayed harvest. Availability of space and nutrient might influence the RGR in the first harvest compared to the successive harvests. The OMs are rich sources of nitrogen (N), phosphorous (P_2_O_5_), and potash (K_2_O) and are usually applied in agricultural land in India (Gaur et al., 1990). The amount of nutrients of OM varied with season and geographical location. The mixture of these three manures fulfills the requirements of the plant. The decomposition of these manures enhances their availability to the plant. In the present study, mixture of manures was decomposed for 5 days before application in the water bodies.

Porath et al. (1979) reported the RGR of fresh *L. minor* cultured in both laboratory and field conditions. They obtained the highest value of 0.346 g/g/day in laboratory condition, whereas the value became 0.099 g/g/day in the field condition. The fresh yield of duckweed ranged from −0.026 to 0.66 Kg/m^2^/week during 8 successive weeks of culture in the manured pond. Oron (1994) reported that the RGRs of duckweeds ranged from 0.10 to 0.35 g/g/day. RGR of *Lemma gibba* grown in desert ponds ranged from 0.081 to 0.191 g/g/day (Guy et al., 1990). Rejmankova (1975) observed 0.20 and 0.22 g/g/day RGR of *L. minor* and *L. gibba*, respectively, in field conditions. In the present study, RGR of duckweed was comparable with the earlier studies.

Hassan and Chakrabarti (2009) reported that variations in climatic conditions, nutritional status of the water body and differences in species resulted in the differences in the production of the macrophytes. Most of the data were generated from short-term studies in small-scale experimental systems. The data generated from longer duration study in commercial-sized systems are most wanted. In the present study, 702.5 Kg (dry mass)/ha/month (i.e., 8.43 tons/ha/year) *L. minor* were produced from pond with the application of OMs. In UASB effluent and nutrient non-limiting water, *L. minor* production were 10.7 and 16.1 tons (dry mass)/ha/year (Reddy and DeBusk, 1985; Vroon and Weller, 1995). In septage-fed pond, the production of *Lemna perpusilla* was 11.2 tons (dry mass)/ha/year (Edwards et al., 1990). It was suggested that in an aquatic environment with sufficient nutrients and optimum environmental conditions around 10–20 tons (dry mass)/ha/year duckweeds can be harvested (Hassan and Chakrabarti, 2009). Seeding of the plant also played important role in the production. DWRP (1998) recommended 40 Kg/100 m^2^ for *L. minor* in order to obtain a dense cover in 3 days. But getting this amount of *L. minor* from control culture system for seeding was difficult. Therefore, only 1 Kg (wet weight) of plant was seeded in the 200 m^2^ pond in the present study. The growth rate of *L. minor* was 3–6 folds higher at the time of first harvest compared to the second and third harvests. Optimum water quality parameters *viz*. temperature, ammonia, phosphate levels etc. influenced the growth of the macrophyte. In the month of July, the presence of moisture in the air also influenced the macrophytes production (compared to the dry season). Moreover, there was enough space at the initial phase; due to the growth of the plants, there was competition for space and nutrients at the later phase.

The impact of various water quality parameters on the production of *L. minor* was documented in the present study. Temperature is known to be the master abiotic factor. In an outdoor facility, *L. minor* were cultured under a wide range of temperature of 11.5–28.4°C during September 2016–April 2017. In the OM-based culture system, production was greatly reduced as the water temperature become < 18.5°C from November onwards. An increasing trend in duckweed production was recorded from February onwards as temperature rose to 19.4°C. In pond the experiment, 31.5–30.3°C temperature was favorable for duckweed production. Temperature tolerance and optima are species-specific. Maximum growth for most of the species of *L. minor* was obtained between 17.5 and 30°C (Culley et al., 1981; Gaigher and Short, 1986). The growth rate declined at low temperature. Reduced growth rate was found in some duckweed at the temperature below 17°C (Culley et al., 1981). Most species seemed to die at 35°C water temperature. All these studies showed that 17–18°C was the critical temperature and 27–31°C was optimum temperature for the production of *L. minor*.

The intensity of light also played major role in the production of *L. minor*. In the experiment 2, as light intensity reduced, particularly in the second and third phases (718–2,138 lux), production reduced drastically; an increasing trend was recorded as light intensity increased (3,463–3,728 lux) in experiment 2. In the present study, maximum *L. minor* production was recorded at 7,353–10,878 lux light intensity. Mkandawire and Dudel (2007) suggested 4,200–6,700 lux light intensity at 14–16 h photoperiod for the optimum production of *L. gibba* and *L. minor*.

Duckweeds have wide range of pH tolerance. The biomass of duckweeds doubled in 2–4 days at pH 7–8 (Culley et al., 1981). Khondker et al. (1994) showed that pH 6.9–7.8 was suitable for *L. perpusilla* production. The optimum growth of *L. perpusilla* was found at pH 7.36 (Van der Does and Klink, 1991). In the present study, pH ranged from 7.32 to 8.04 in all outdoor and pond culture systems in OM and IF. Application of higher dose of OMs resulted in pH < 7.0 in 2x OM and 2x OM+IF in experiment 1. The pH of water decreased after the application of OMs in all experiments and then increased with the duration of study.

In the production of *L. minor*, a direct effect of dissolved oxygen was not recorded. In OM, dissolved oxygen level was generally < 1.0 mg/l, whereas a higher concentration of dissolved oxygen was always recorded in IF. But the production was lower in IF compared to OM in most of the harvestings (except third phase of experiment 2). Application of OMs reduced the oxygen level in the culture system. Dissolved oxygen might influence the nitrification of ammonia as higher level of nitrite and nitrate levels were found in IF compared to OM.

Ammonia (NH_3_) level was always lowest in IF regardless of experiment. Leng et al. (1995) suggested that ionized ammonia (${\text{NH}}_{4}^{+}$) as preferable nitrogenous substrate for *L. minor* culture. The ammonium-ammonia balance shifted toward the un-ionized (NH_3_) form at alkaline pH. This resulted in higher concentration of free ammonia in the culture system which affected the duckweed production. Leng et al. (1995) also suggested that the ammonia concentrations of cultured water should be 7–12 mg N/l for the maintenance of crude protein content of duckweed. In the present study, higher level of ammonia (18.8–32.0 mg/l) also affected the production of duckweed in culture systems fertilized with OMs, especially during winter. In experiment 2, significantly (*P* < 0.05) higher level of ammonia (combined with low temperature) affected the growth of macrophytes in OM+IF treatment. Porath and Pollock (1982) suggested that ammonia (${\text{NH}}_{4}^{+}$) uptake is temperature-sensitive in duckweed. Nitrite and nitrate levels were lowest in OM. Higher levels of nitrite and nitrate in IF showed the better nitrification rate in this treatment compared to the OM. Duckweeds preferred ammonia as nitrogen source compared to nitrate and grew better in presence of the former nutrient (Lüönd, 1980).

Phosphorus is one of the limiting nutrients (after nitrogen) and is essential for rapid growth of the macrophyte. In OM, phosphate level was most of the time >1.0 mg/l, whereas phosphate level was < 1.0 mg/l in IF. This showed the phosphate limitation of this treatment. In some species of duckweeds, decreased growth rate was obtained at *P*-values < 0.017 mg/l (Lüönd, 1980). In *L. perpusilla*, a positive correlation was recorded between the concentrations of phosphate and silicate and the biomass (Khondker et al., 1994). Phosphorous (PO_4_-P) should range between 4 and 8 mg/l for the optimum production of duckweeds (Hassan and Chakrabarti, 2009). The present study showed that the production of *L. minor* was dependent on nutrient availability in terms of nitrogen and phosphorous and environmental factors like temperature and light intensity of the culture system.

Many studies showed the proximate composition of several species of duckweeds from different geographical areas. Protein content ranged from 14.0 to 23.5, 25.3 to 29.3, 9.4 to 38.5, and 26.3 to 45.5% in *L. minor* (Majid et al., 1992; Zaher et al., 1995), *L. perpusilla* (Hassan and Edwards, 1992), *L. gibba* (Hillman and Culley, 1978; Culley et al., 1981), and *Lemma paucicostata* (Mbagwu and Adenji, 1988), respectively. In the present study, 36.07 ± 0.18 and 27.12 ± 0.4% protein contents were found in *L. minor* cultured in OMs and IFs. The nutritional status of the water body influenced the crude protein content of the duckweed; protein content ranged from 9 to 20% in nutrient-poor water or under sub-optimum nutrient conditions, whereas it ranged 24–41% in nutrient-rich water. The crude protein content of duckweed increased up to 40% at ammonia concentration of 7–12 mg N/l (Leng et al., 1995). The protein content of *L. minor* collected from a natural pond of northeast region of India was 28.0 ± 1.7 (Kalita et al., 2007). Appenroth et al. (2017) reported that in different species of duckweeds protein contents ranged from 20 to 35%.

Presence of high quality protein was reported in various studies (Porath et al., 1979; Rusoff et al., 1980). The essential amino acid profile of duckweed was better compared to the most of the plant proteins and more closely resembled to animal protein. Guha (1997) reported that the protein of duckweeds was rich in certain amino acids that were often low in plant proteins. The nutritional value of duckweeds is comparable with alfalfa in terms of two essential amino acids—lysine and arginine. These are required in animal feeds. High amount of leucine, threonine, valine, isoleucine, and phenylalanine and less amount of methionine and tyrosine are found in duckweeds. The amino acid content of the *L. paucicostata* was equivalent to that of blood, soybean, and cottonseed meals and considerably exceeded that of groundnut meal (Mbagwu and Adenji, 1988). Amino acids like, lysine (4.8%), methionine and cystine (2.7%), and phenylalanine and tyrosine (7.7%) were also present in the duckweeds (Appenroth et al., 2017). In the present study, the amino acid composition of *L. minor* confirmed its nutritional value as feed ingredient as it is a rich source for essential and non-essential amino acids. The presence of non-proteinogenic amino acids viz. taurine, citrulline, hydroxyproline, sarcosine etc. enhanced the nutritional value of duckweed.

Like protein, lipid, and ash contents of *L. minor* grown in OM were higher compared to the macrophytes grown in IF in the present study. Culley et al. (1981) reported 6.3% ether extracts in *L. gibba* from USA. In different species of duckweeds fat contents ranged from 4 to 7% (Appenroth et al., 2017). Lipid content of *L. minor* was higher (7.15–8.45%) in the present study, but slightly lower than the 10.6% reported by Yan et al. (2013). The duckweeds produced in nutrient poor water bodies showed lower lipid content (1.8–2.5%) compared to the plant grown (3–7% lipid) in water enriched with nutrient (Hassan and Chakrabarti, 2009). The lipid and ash contents of *L. minor* grown in natural pond were 5.0 ± 0.1 and 25.0 ± 1.6%, respectively (Kalita et al., 2007). Ash contents of various species of *L. minor* ranged from 11.1 to 17.6% (Hassan and Edwards, 1992; Zaher et al., 1995). The ash content of *L. minor* ranged from 19.42 to 21.41% in the present study. Duckweeds are known to accumulate large amounts of minerals in their tissues. Higher amount of ash and fiber and lower amount of protein were found in duckweed colonies with slow growth rate (Skillicorn et al., 1993). The ash content of duckweed was not influenced by the nutrient status of water (Leng et al., 1995). The fatty acid content of *L. minor* cultured in the present study was generally similar to that measured by Yan et al. (2013), who reported a composition of around 25% saturated fatty acids, 5% monoenes and 70% PUFA, with a very similar level of LA (16%) but a higher proportion of LNA at ~54% of total fatty acids. The trace level of EPA found in the present study most likely simply reflects the presence of a very small amount of freshwater microalgae, which can often contain some EPA but rarely DHA, in the *L. minor* harvest. Negesse et al. (2009) also reported the presence of short chain fatty acids (SCFA, 16.6%) in *L. minor* (C2 11%, C3 3.1%, C4 1.4%, and C5 0.4%) that could be useful for commercial utilization of duckweed as they can serve as preservatives, preventing bacterial growth. In recent study, Appenroth et al. (2017) reported that in different species of duckweeds, polyunsaturated fatty acids content ranged from 48 to 71%; the high level of n3 fatty acids resulted in a favorable n6/n3 ratio and enhanced the nutritional value of the duckweeds.

## Conclusions

The present study showed that *L. minor* can be produced using cheap and easily available OMs and the produced macrophytes are rich source of protein, lipid, and minerals. Amino acid profile and fatty acid profile confirmed the suitability of the macrophytes in the production of aqua-feed.

Among water quality parameters temperature, light intensity, pH, ammonia, phosphate, and conductivity played major role. These factors should be maintained within reasonable limits for survival and growth of the macrophytes. The management strategies for duckweed culture should focus on the time of manure application and harvesting.

## Materials and methods

### Culture of *L. minor* in outdoor tanks

*Lemna minor* was collected from a pond located in the Department of Botany, University of Delhi and the plant was identified based on the morphological characteristics (oval shaped fronds, 2–5 fronds remained together, presence of three nerves in each frond and cylindrical root sheath with two lateral wings) with the help of scientist of Department of Botany. Since then the macrophyte was cultured in the Department of Zoology. Three consecutive experiments were conducted to generate the baseline data for the production of *L. minor*. Macrophytes were grown in cemented tanks (1.2 × 0.35 m) maintained in the outdoor facility of Department of Zoology, University of Delhi. The depth of water was 30 cm throughout the study period. Dechlorinated tap water, supplied by the Municipal Corporation of Delhi was used for all experiments. The first experiment was conducted during September–October 2016 and the duration of the experiment was 30 days. Four different manures were used. In manure 1 (OM) and manure 3 (OM 2x OM), cattle manure (local), poultry droppings (local), and mustard oil cake (Double Hiran Mustard Oil-cake, Malook Chand Food Pvt. Ltd., Aligarh, U.P., India) (1:1:1) were used at the rate of 1.052 and 2.104 Kg/m^3^ (Srivastava et al., 2006); in manure 2 (IF), urea (IFFCO, Indian Farmers Fertilizer Cooperative Limited, New Delhi, India), potash (Narmada, Gujarat Narmada Valley Fertilizers & Chemicals, Gujarat, India), triple superphosphate (IPL, Indian Potash Limited, Chennai, India) were used at the rate of 15, 3, and 3 Kg/ha/day, respectively, based on the study of DWRP (1998); manure 4 (2x OM+IF) was a combination of manure 2 and manure 3. The amount of IFs was calculated for 10 days and applied in the culture tank. In all these experiments, OMs were applied at the rate of one fourth dose of initial dose at every 10 days interval. In IFs, similar dose (initial amount) of manures was applied at every 10 days interval. Manures for individual tank were mixed with tap water and allowed to decompose for 5 days before application. All manures, except cattle manure were applied in dry conditions. The moisture content of cattle manure was measured and the weight was adjusted.

The second experiment was conducted during October 2016–February 2017 and the duration of the experiment was 105 days. The three different manures used in this experiment were selected based on the results of the first experiment. The first two manures, manure 1 (OM) and manure 2 (IF) were similar to the earlier experiment, and manure 3 (OM+IF) was a combination of manure 1 and manure 2. Third experiment was conducted during February–April 2017. In this experiment, manure 1 (OM) and manure 2 (IF) were selected. This selection was based on the production potential of these manures compared to the others.

Fresh *L. minor* (15 g wet weight) was seeded after 5 days of manure application in each tank. Three replicates were used for each treatment. Harvesting of *L. minor* started when the plant covered the whole surface area of the tank; 50% of the total production was harvested during first and other consecutive harvests; all macrophytes were harvested at the end of the study. Production was expressed as Kg/m^2^/month on wet weight basis.

### Culture of *L. minor* in the ponds

**Figure 11:**
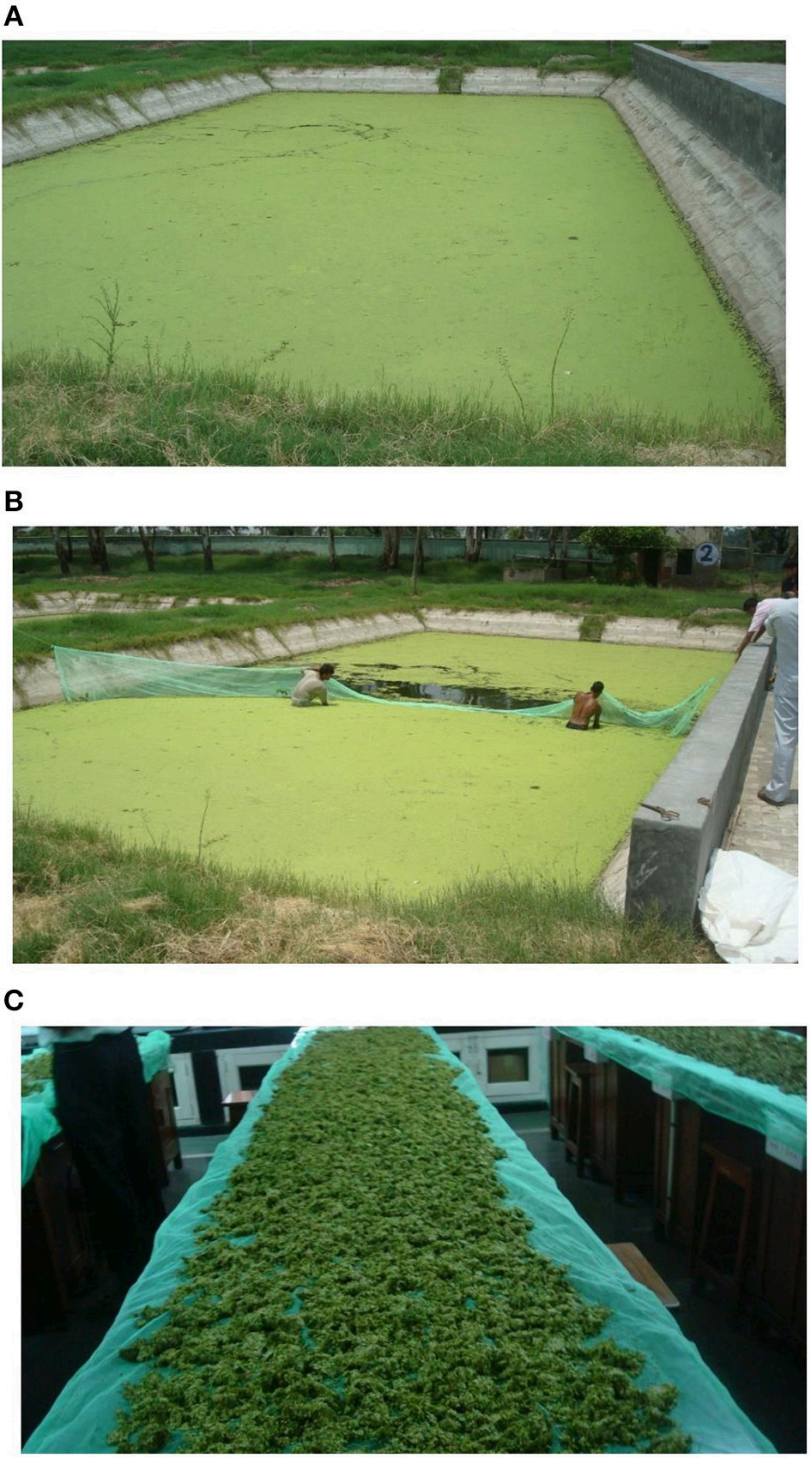
**(A)** Production of *L. minor* after 10 days of culture in the pond of Central Institute of Fisheries Education, Rohtak, Haryana, India. **(B)** The harvesting (50%) of *L. minor* in pond. **(C)** Air drying of harvested duckweed at University of Delhi.

In CIFE, Rohtak Center (Indian Council of Agricultural Research), Haryana three cemented ponds (200 m^2^, 20 m × 10 m) were prepared for the culture of *L. minor* in June 2017. The bottom was cleaned thoroughly and the pond was filled with ground water (50 cm). The OMs, like cattle manure, poultry dropping, and mustard oil cake (1:1:1) were applied at the rate of 1.052 Kg/m^3^ (Srivastava et al., 2006). Organic manures applied for the production of duckweed were selected based on the results of outdoor cemented tanks. Three replicates were used for the study. The culture conditions of *L. minor* developed in the tank experiments were also applied in the pond. All manures were mixed properly and allowed to decompose for 5 days. Then fresh *L. minor* cultured in cemented tanks of Department of Zoology, University of Delhi was seeded (wet weight) at the rate of 1 Kg/pond. It covered a small area of the pond. The experiment was continued for 30 days. Like outdoor tank experiments, one fourth dose of initial dose of manure was applied at every 10 days interval. Harvesting started when the plant covered the whole surface area of the pond. Total pond area (200 m^2^) was divided in four quadrates of 50 m^2^ each. In first and second harvests, *L. minor* was collected from two quadrants for 50% harvesting (Figures 11A,B). In third harvest, all plants were collected and air dried (Figure 11C). Production was expressed as Kg/m^2^/month on wet weight basis.

### Water quality

Various water quality parameters were monitored regularly in outdoor tanks and in ponds at 9.00 a.m. Water temperature and pH (PHC 10101), conductivity (CDC 40101), dissolved oxygen (LDO 10101), ammonia, NH_3_ (ISENH318101), and nitrate (ISENO318101) were measured using HACH multimeter (HQ 40d, USA). Light intensity was measured with probe (PMA 2130) attached with a lux meter (SOLAR LIGHT, PMA 2100, USA) at the surface of the water. Phosphate (4500-P D. Stannous Chloride Method) and nitrite (4500-${\text{NO}}_{2}^{-}$ B. Colorimetric Method) were measured following the method of APHA (2012).

### Relative growth rate

The RGR of *L. minor* was calculated using the formula: RGR = ln (*W*_*t*_*/W*_0_)/*t*.

Where, *W*_*t*_ and *W*_0_ are the fresh weight of macrophytes at the time of harvest (*t*) and at the time of introduction of plant (zero reference time), respectively; *t* is the time interval in days.

Fresh *L. minor* was used for the study and RGR-value was expressed as g/g/day, i.e., production (g) of *L. minor* from 1 g of starter culture of *L. minor* per day.

### Composition of *L. minor*

Biochemical (proximate) compositions of *L. minor* cultured in tanks (OM and IF) were determined by standard methods (AOAC, 2000). Moisture contents were recorded after drying at 110°C for 24 h, and the ash fraction obtained after incineration at 600°C for 16 h. The crude protein content was determined by measuring nitrogen content (N × 6.25) by Kjeldahl analysis (Tecator Kjeltec 1030 analyser, Foss, Warrington, UK). The crude lipid content was measured gravimetrically after extraction by Soxhlet (Tecator Soxtec 2050, Warrington, UK). Carbohydrate content was then determined by the subtraction method.

Amino acid composition was estimated using Automatic Amino Acid Analyzer L-8900 (Hitachi Co. Ltd., Tokyo, Japan). Dry and powdered plant sample was hydrolyzed with 6 N HCl at 110°C for 22 h. Hydrolyzed sample was dried in Nitrogen Evaporator (PCi Analytic Private Limited, Maharashtra, India). Then 0.02 N HCl was added in the sample and concentration of protein in the sample was 0.5 mg/ml. The sample was kept in the Auto sampler. Sample injection volume was 20 μl. Methionine, cysteine, and tryptophan are destroyed during hydrolysis with 6 N HCl; these amino acids are treated with specific reagents. Methionine and cysteine were oxidized with performic acid and then treated with 48% hydrobromic acid. Macrophyte sample was hydrolyzed with 4 N methanesulfonic acid and 3-(2-aminoethyl) indole for the estimation of tryptophan. Rest of the methods were same for all amino acids. The ninhydrin derivative of proline and hydroxyproline was monitored at 440 nm, and other amino acids were monitored at 570 nm. The contents of detected amino acids were quantified by comparing their peak areas with those of authentic standards provided with the equipment. Amino Acids Mixture Standard Solutions, Type B and Type AN-2 (Wako Pure Chemical Industries, Limited) were used. Standard solutions for glutamine and tryptophan (Sigma-Aldrich, USA) were prepared before analysis.

For fatty acid composition, *L. minor* samples were dried at 40°C and ground. The total lipid fraction was then extracted from 1 g of dried material by homogenization in chloroform/methanol (2:1, v/v) using a tissue disrupter (Ultra-Turrax, Fisher Scientific, Loughborough, UK), and lipid content determined by weighing (Folch et al., 1957). Fatty acid methyl esters (FAME) of total lipid were then prepared by acid-catalyzed transesterification for 16 h at 50°C (Christie, 2003). The FAME were extracted and purified as described in detail previously (Tocher and Harvie, 1988), and then separated and quantified by gas-liquid chromatography (Fisons GC-8160, Thermo Scientific, Milan, Italy) using a 30 m × 0.32 mm i.d. × 0.25 μm ZB-wax column (Phenomenex, Cheshire, UK), on-column injection and ionization detection. Identification of FAMEs was by comparison to known standards and published data (Tocher and Harvie, 1988), and data collected and quantified using Chromcard for Windows (Thermoquest Italia S.p.A., Milan, Italy).

### Statistical analysis

Data were presented as mean ± SE unless otherwise stated. Data were analyzed using one-way analysis of variance (ANOVA), Duncan's multiple range test, DMR (Montgomery, 1984) and, where appropriate, by Student's *t*-test (SPSS software 19.0). Statistical significance was accepted at *P* < 0.05 level.

## Author contributions

RC, DT, and JS designed the study; RG, AS, RC, and JS cultured the plant and analyzed samples; WC and DT analyzed samples; RC, DT, and JS wrote the manuscript; RG, WC, and AS prepared graphs and tables.

### Conflict of interest statement

The authors declare that the research was conducted in the absence of any commercial or financial relationships that could be construed as a potential conflict of interest.
